# Gas Barrier, Rheological and Mechanical Properties of Immiscible Natural Rubber/Acrylonitrile Butadiene Rubber/Organoclay (NR/NBR/Organoclay) Blend Nanocomposites

**DOI:** 10.3390/ma13112654

**Published:** 2020-06-10

**Authors:** Hanna J. Maria, Martin George Thomas, Marco Morreale, Francesco Paolo La Mantia, Ange Nzihou, Kuruvilla Joseph, Didier Rouxel, Susana C. M. Fernandes, Nandakumar Kalarikkal, Sabu Thomas

**Affiliations:** 1 School of Energy Materials, Mahatma Gandhi University, Priyadarshini Hills, Kottayam 686560, Kerala, India; hannavidhu@gmail.com; 2 International and Inter University Centre for Nanoscience and Nanotechnology, Mahatma Gandhi University, International and Inter University, Priyadarshini Hills, Kottayam 686560, Kerala, India; nkkalarikkal@mgu.ac.in; 3 School of Chemical Sciences, Mahatma Gandhi University, Priyadarshini Hills, Kottayam 686560, Kerala, India; 4 Université de Pau et des Pays de l’Adour, E2S UPPA, CNRS, IPREM, 64000 Pau, France; tmmartin@rediffmail.com (M.G.T.); susana.fernandes@univ-pau.fr (S.C.M.F.); 5 Faculty of Engineering and Architecture, Kore University of Enna, Cittadella Universitaria, 94100 Enna, Italy; marco.morreale@unikore.it; 6 Department of Engineering, University of Palermo, 90128 Palermo, Italy; 7 Consorzio INSTM, 50121 Firenze, Italy; 8 IMT Mines, RAPSODEE, 81013 Albi, France; ange.nzihou@mines-albi.fr; 9 Department of Chemistry, Indian Institute of Space Science and Technology, Thiruvananthapuram 695547, Kerala, India; kuruvilla@iist.ac.in; 10 Institut Jean Lamour, UMR 7198 CNRS-Université de Lorraine, F-54500 Vandoeuvre-lès-Nancy, France; didier.rouxel@univ-lorraine.fr; 11 School of Pure and Applied Sciences, Mahatma Gandhi University, Priyadarshini Hills, Kottayam 686560, Kerala, India

**Keywords:** gas permeability, polymer blend, nanoclay

## Abstract

In this paper, gas permeability studies were performed on materials based on natural rubber/acrylonitrile butadiene rubber blends and nanoclay incorporated blend systems. The properties of natural rubber (NR)/nitrile rubber (NBR)/nanoclay nanocomposites, with a particular focus on gas permeability, are presented. The measurements of the barrier properties were assessed using two different gases—O_2_ and CO_2_—by taking in account the blend composition, the filler loading and the nature of the gas molecules. The obtained data showed that the permeability of gas transport was strongly affected by: (i) the blend composition—it was observed that the increase in acrylonitrile butadiene rubber component considerably decreased the permeability; (ii) the nature of the gas—the permeation of CO_2_ was higher than O_2_; (iii) the nanoclay loading—it was found that the permeability decreased with the incorporation of nanoclay. The localization of nanoclay in the blend system also played a major role in determining the gas permeability. The permeability of the systems was correlated with blend morphology and dispersion of the nanoclay platelets in the polymer blend.

## 1. Introduction

The improvement of the barrier properties of polymer blends is profitable for the packaging or coating industries, namely pharmaceuticals and the packaging of electronic items and food products, which are sensitive to gaseous molecules. This can be achieved by combining an elastomer with poor barrier properties with a highly impermeable elastomer, preferably, if it can be produced by an industrially viable method. Blending incompatible polymers often results in poor dispersion, in which the dispersed phase is very large, and there is weak interface adhesion between the two polymers. The blend morphology influences its transport properties to a great extent [1,2]. For instance, Zembouai et al. [3] studied the barrier properties of poly (3-hydroxybutyrate-co-3-hydroxyvalerate)/polylactide (PHBV/PLA) blends prepared by melt mixing. They reported that PHBV imparted better water and oxygen barrier properties to PHBV/PLA blends by acting as an efficient barrier promoter for PLA, even at quite a low ratio. Additionally, Lafitte et al. [4] have studied the influence of the blend composition and morphology on the barrier properties of polyamide 11/poly (hydroxy amino ether) blend. They observed an improvement of hydrogen barrier properties and related it to the blend composition. Moreover, a significant effect of the blend morphology was observed on mechanical properties in the rubbery state. Subramanian et al. [5,6] have studied the barrier properties of polymer blends and reported on the influence of morphology. They reported that in polymer blends polymers dispersed as essentially parallel, thin, large laminae produce substantial reduction (3–100 times) of permeability properties in blow-molded/extruded samples.

However, in many cases, the required properties cannot be reached in incompatible polymer blends, because of the weak adhesion and presence of voids or free volume. The major problems of the incompatible polymer blends can be reduced to a great extent by incorporating a compatibilizer agent. In immiscible polymer blends, compatibilizers can improve the interface and modify the dispersion, reduce the interfacial tension, suppress coalescence and/or influence other parameters like viscosity, which can contribute to the homogenous dispersion of the minor phase. This will lead to excellent blend mechanical properties and transport properties. Thus, by carefully controlling the blend morphology, many blend properties, including the gas transport through polymer blends, can be modified. This is mainly influenced by blend composition, nature of blend components and the presence of other materials, such as fillers and additives. The introduction of nanoparticles can impart some significant effect in tuning up the blend morphology [7]. Recently, some studies have been reported [8], which have made use of nanoparticles as property enhancer or compatibilizer agents in immiscible polymer blends [9,10,11]. Frounchi et al. [12], have studied the gas barrier properties of polypropylene/ethylene-propylene-diene-monomer(PP/EPDM) blend nanocomposites, and found that the oxygen and carbon dioxide permeability of the nanocomposite reduced twice by adding only 1.5 vol % of nanoclay. Yeh et al. [13] have investigated the oxygen barrier properties of clay mineral nanocomposites prepared from modified polyamide (MPA) and nylon-6 clay (NYC) blends, and found that at 20 wt % optimum content of NYC, the oxygen barrier improvement of nanocomposites reached the maximum. [14] All films have been shown to possess superior oxygen barrier properties compared to the plain polyethylene (PE) films. Ghanbari et al. [15] studied the O_2_ barrier properties of polymer/organoclay nanocomposites based on poly(ethylene terephthalate) (PET) and reported that, for all the nanocomposite films, in comparison to a neat PET, the permeability is decreased due to both the presence of clay particles and a higher crystallinity. This proved strong influence of the nanoclay distribution on the barrier properties. Bitinis et al. [16] have studied the barrier properties of organoclay filled polylactic acid/natural rubber blend bionanocomposites and observed that organoclays were preferentially located at the interface and acted as a compatibilizer agent between both polymer phases and resulted in a marked improvement of the physical and mechanical properties of the system.

In this context, the purpose of this paper is to have a deep understanding of the effect of nanoclay in enhancing the gas barrier properties of immiscible and incompatible natural rubber/acrylonitrile butadiene rubber (NR/NBR) blends. The study also aimed in establishing the relationship between barrier properties and morphology the blends and blend nanocomposite systems. Herein, it is reported that the analysis of gas transport behaviour taking in account the blend composition, the filler loading and the nature of permeant. The paper will also focus on explaining the rheological and mechanical property changes by the incorporation of nanoclay. To our best knowledge, no detailed studies have been reported so far on NR/NBR blend system explaining the effect of morphology, nature of gas and filler loading on the gas barrier properties, mechanical behaviour and rheological characteristics. This elastomeric blend with organically modified nanoclay, Cloisite 10A, has not been widely studied with a focus on its application as barrier material. This paper is very significant for the design of new materials and also in future analysis (both experimental and theoretical work) related to the area.

## 2. Materials and Methods

### 2.1. Materials

Natural rubber (NR, ISNR 5) was supplied by the Rubber Board, Kottayam, India, and has a number average molecular weight of 3 × 10^5^ g/mol, a weight average molecular weight (Mw) of 7.8 × 10^5^ g/mol, [17] and a Mooney viscosity 85 mL (1 + 4) at 100 °C. Nitrile rubber with 33% acrylonitrile content (NBR, Chemigum^®^ N344) with a Mooney viscosity of 38–45 mL (1 + 4) at 100 °C and specific gravity of about 0.98 was supplied by Eliokem industries Ltd. Mumbai, India. The organically modified montmorillonite used in this study was Cloisite 10A (montmorillonite with organic modification dimethyl, benzyl, one alkyl tail hydrogenated Tallow (HT, 65 m% C18, 30 m% C16, 5 m% C14) modification provided by Southern Clay Products (USA). The cation exchange capacity (CEC) was equal to 125 meq/100 g, and an average dry particle size in the range 2–13 µm.

### 2.2. Preparation of the Blend Nanocomposite

**Scheme 1:**
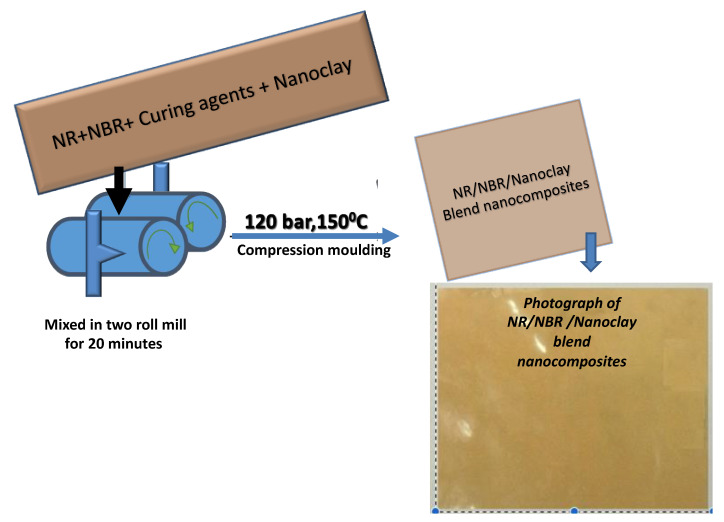
Flow chart showing the preparation of natural rubber/acrylonitrile butadiene rubber (NR/NBR) blend nanocomposites.

The NR/NBR nanocomposite were prepared according to the formulation given in (Table 1) by blending in a laboratory-sized two roll mixing mill. The NR/NBR blends were compounded according to ASTM D 3182 (Standard practice for rubber). The two rubbers were mixed using a laboratory-sized two-roll mill (Kelachandra Machines in Chingavanam, Kottayam-Local manufactures), with a roll diameter (OD) of 150 mm, and a roll length of 300 mm, with a friction ratio of 1:1.25, as per ASTM D 3184-89. The mill opening was set at 1.4 mm, and the initial temperature of the mill was set at 80 ± 5 °C. The curing agents were added to the blended rubber, followed by ¾ cuts on both sides of the band, tailed by the incorporation of accelerator and vulcanizing agents (CBS, TMTD and sulfur) for a time period of 2.0 min each was done. For all the mixes the nip gap, roll speed ratio, and the number of passes were kept constant. The temperature range for mixing was 70–90 °C. After mixing, the rubber compositions were molded in an electrically heated hydraulic press to the optimum cure, using the molding conditions explained below. The samples were then compression molded at 150 °C, with a cure time obtained from an oscillating disc rheometer (RheolineMulti-Function Rheometer-manufactured by Tewkesbury, UK, supplied by Vaibhavi International Trading House) at 150 °C for 15 min according to ASTM D2084. The composites were cured at their respective cure times in a hydraulic press, under a pressure of about 120 bar at 150 °C, and was made into a 1 mm sheet. (Scheme 1). Round shaped samples of 1 mm of thickness were used for the diffusion study. All the blend compositions from now on will be represented in the order NR/NBR. The different NR/NBR compositions prepared were in the 100/0, 70/30, 50/50, 30/70 and 0/100 ratios. The specimens for various tests were taken in accordance with ASTM standards.

For the preparation of the nanocomposites the same method was used as above and for each blend composition 1 phr, 2 phr, 5 phr and 10 phr nanoclay were added soon after the addition of activators zinc oxide and stearic acid. This was followed by ¾ cuts on both sides of the band, tailed by the incorporation of accelerator and vulcanizing agents (CBS, TMTD and sulphur) for a time period of 2.0 min each. The details of the composition are given in Table 2.

### 2.3. Characterisation

#### 2.3.1. Gas Permeability

The gas permeability of the nanocomposites was measured using Lyssy Manometric Gas Permeability Tester L100-2402 (Illinois Instruments Inc., Johnsburg, IL, USA). The test gases used were oxygen, and CO_2_ at a rate of 500 mL/min. The test was conducted at a temperature of (25 ± 1) °C and a relative humidity of 65 ± 0.5%. The thickness of the sample was 1 mm.

#### 2.3.2. Rheological Analysis

The rheological measurements were performed on a stress-controlled rheometer (REOLOGICA Instruments AB, Lund, Sweden). Frequency sweep measurements were carried out over a frequency range of 0.01 Hz−40 Hz at 100 °C. The complex viscosity (η*) was measured in the frequency sweep experiments performed with data collected at ten points per decade and the strain amplitude was 1%.

#### 2.3.3. Mechanical Analysis

Mechanical properties of the samples were measured using universal testing machine (T50KT, Tinius Olsen, Horsham, PA, USA) with a crosshead rate at 500 mm/min, according to ASTM D 412 at a room temperature (25 ± 2 °C). In particular, five dumbbell shaped samples of each system were used to determine the Young’s modulus.

#### 2.3.4. Scanning Electron Microscopy

The cryogenically fractured surface and smooth cut surface of the blend samples were investigated using an environmental scanning electron microscope (PHILIPS XL30 ESEM FEG, FEI Philips, Eindhoven, The Netherlands), with an accelerating voltage of 8 kV.

#### 2.3.5. Transmission Electron Microscopy

To assess the quality of filler dispersion and morphological details, the NR/NBR nanocomposites were investigated by means of transmission electron microscopy (TEM) (JEM-2100 HRTEM, Jeol, Tokyo Japan). The micrographs were obtained in point to point resolution 0.194 nm, operating at an accelerating voltage of 200 kV. Ultrathin sections of bulk specimens Cryocut specimens (~100 nm thickness) prepared using an ultra-microtome (Ultracut UCT, Leica, Wetzlar, Germany) were placed on a 300 mesh Cu grids (35 mm diameter) and were analyzed without staining.

## 3. Results and Discussion

### 3.1. Effect of NR/NBR Blend Composition on Oxygen Permeability

**Figure 1:**
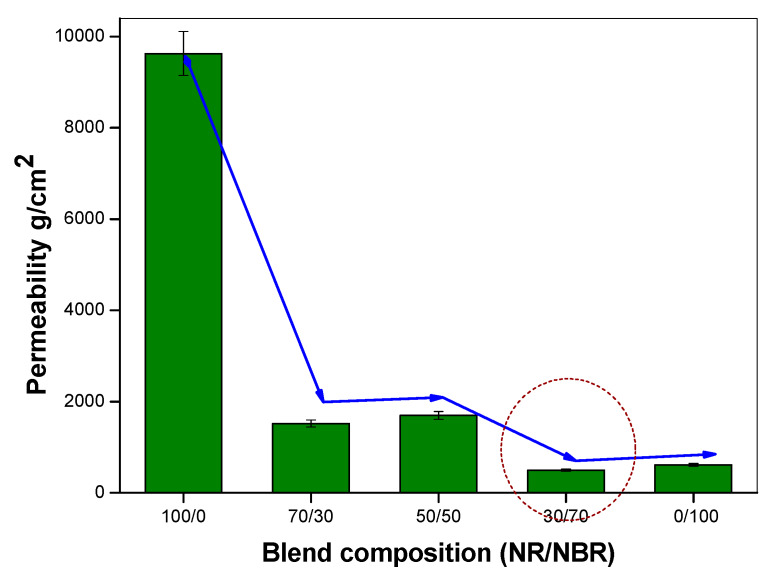
Oxygen permeability of different NR/NBR blends with varying blend compositions.

To assess the effect of the NR/NBR blend composition on oxygen barrier properties different blend compositions (without nanoclays) were considered, as described in Table 2. The data described in Figure 1 show a considerable decrease on O_2_ gas permeability with the increasing NBR content. It can be first observed that barrier properties of oxygen through NR/NBR blends show improvement upon increasing the NBR content. The gas permeability values, shown in Figure 1, strongly indicate the extent of improvement compared to neat NR without nanoclay. NR is highly permeable to oxygen. Contrarily, NBR has very high resistance to oxygen transport due to it polar groups. Interestingly, it can be noted that for 30/70 (NR/NBR) composition, 94% of improvement in gas impermeability was observed (improved about 16-fold) by adding 70 wt % of NBR to NR, while for the 70/30 and 50/50 NR/NBR blends, the impermeability was increased by 84% and 82%, respectively. The 30/70 NR/NBR composition is found to be slightly better than neat NBR. This behavior could be correlated to the morphology of the blend systems.

**Figure 2:**
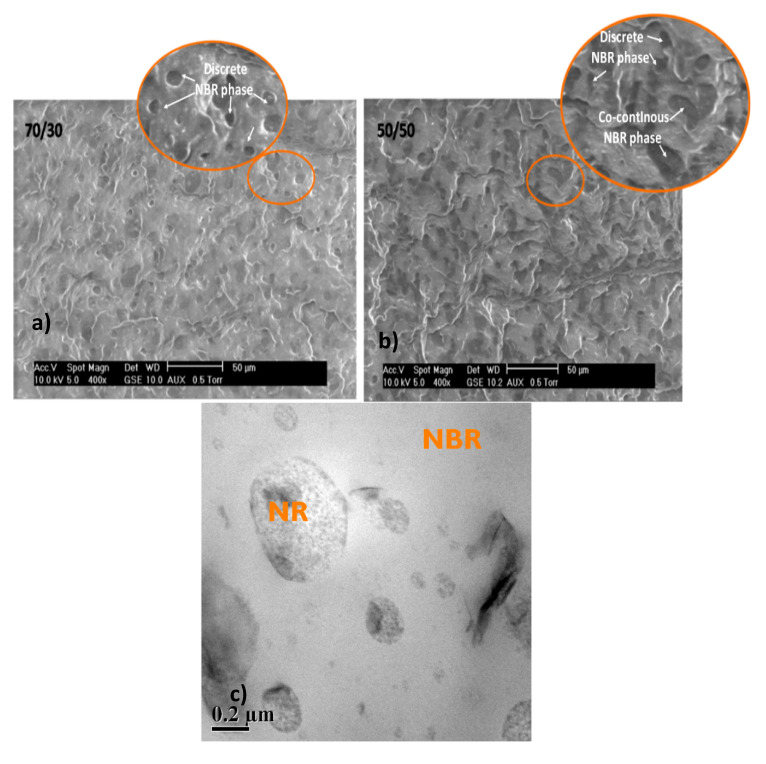
SEM images of (**a**) 70/30 NR/NBR blend with dispersed morphology, (**b**) 50/50 NR/NBR blend with co-continuous morphology, (**c**) transmission electron microscopy (TEM) image of 30/70 NR/NBR blend nanocomposites with dispersed morphology.

The morphology of the blends 70/30 and 50/50 NR/NBR is shown in Figure 2a,b. In the case of 70/30 NR/NBR blends, the NR is represented by the continuous phase and the NBR is represented by a discontinuous domain represented by discrete dispersed NBR phases. For the 50/50 blends, both phases tend to be continuous phases; interestingly, the NBR domains have two different morphologies: discrete dispersed and co-continuous phases. Considering NBR as a matrix phase for the 30/70 NR/NBR system, the NBR is the continuous phase where NR becomes the dispersed phase (Figure 2c). In general, the SEM micrographs show that the blends are heterogeneous in nature. The lowest permeability value of the 30/70 NR/NBR blend is associated with the continuous nature of the NBR phase.

In fact, this blend composition has given the best impermeability values among all the blend nanocomposites systems. The permeability of a gas molecule through a polymeric membrane can be determined from the relationship between cohesive energy density and activation energy given by the equation developed by Meares Equation (1) [18].

(1) $$\;\;\;\;E_{D}=\pi /\left(4\;\sigma^{2}\;\lambda N_{A}\left(CED\right)\right)$$

where *E_D_* is the activation energy of diffusion, *σ*^2^ is the cross section of the penetrant molecule, *λ* is the jump length, *N_A_* is the Avogadro’s number and CED is the cohesive energy density. The polarity of the NBR molecules makes the cohesive energy density of NBR high and hence results in low permeability. The reason for the decrease in permeability on adding NBR can thus be clearly explained. The Meares equation thus shows the relationship between various transport parameters like diffusion coefficients, permeability coefficients, activation energy of diffusion (ED) and cohesive energy density (CED) of the polymers. It explains that activation energy of diffusion is proportional to the CED of a polymer in which diffusion takes place, which implies that the diffusion coefficient and also the permeability coefficient due to the relation are both affected by the CED [19,20].

### 3.2. Effect of Filler Loading on Oxygen Permeability

**Figure 3:**
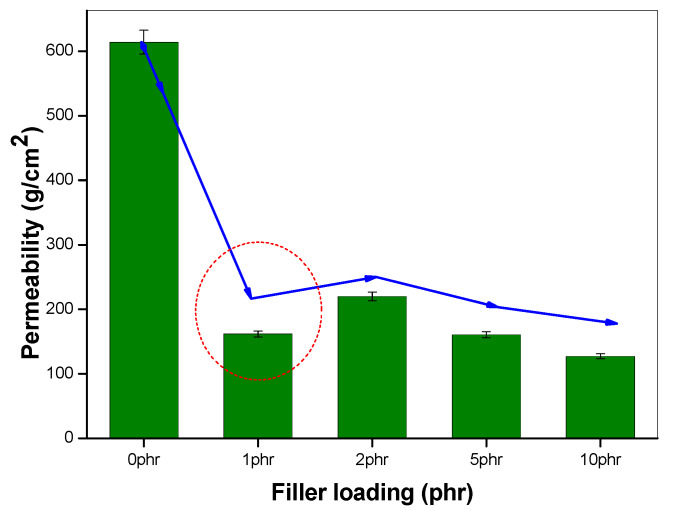
Oxygen permeability of NBR nanocomposites, with varying filler loading.

**Figure 4:**
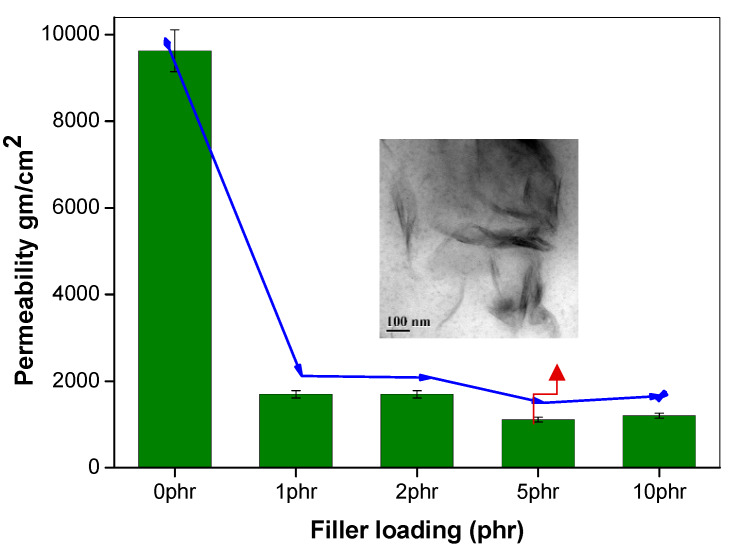
Oxygen permeability of NR nanocomposites by varying filler loading; (inset) the TEM image of NR nanocomposite at 5 phr loading.

**Figure 5:**
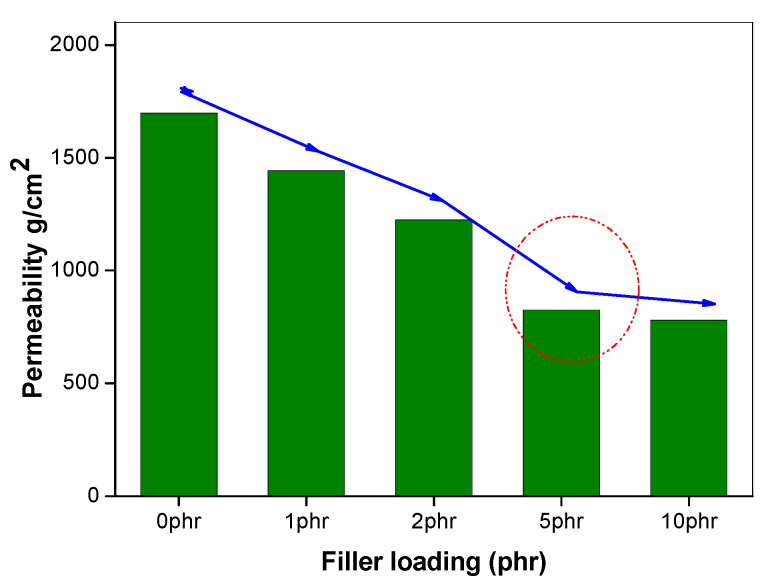
Oxygen permeability of 50/50 NR/NBR blend by varying filler loading.

**Figure 6:**
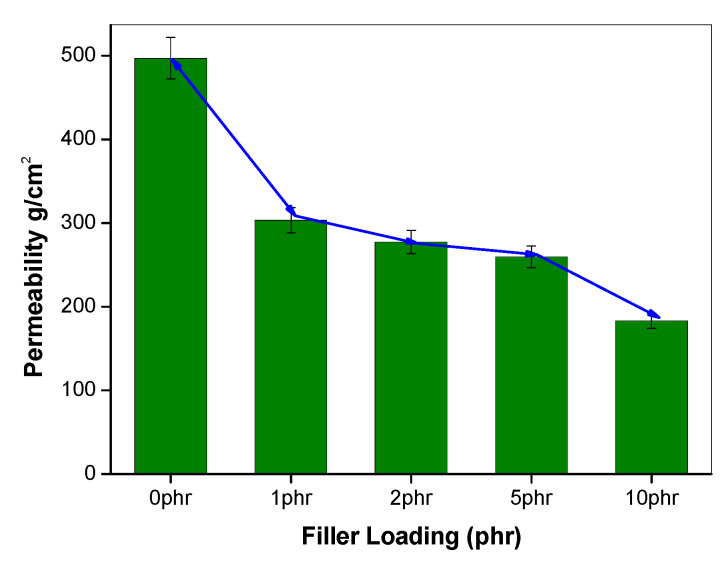
Oxygen permeability of a 30/70 NR/NBR blend with varying filler loading.

**Figure 7:**
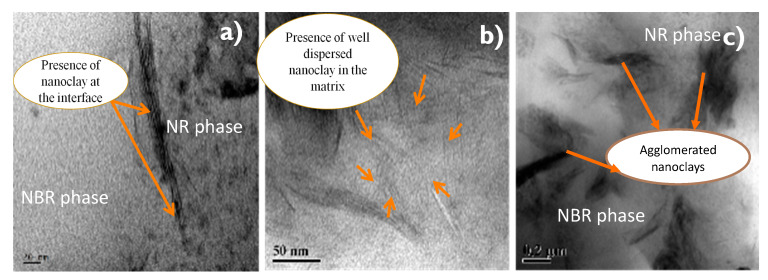
(**a**–**c**) TEM images showing of 50/50 NR/NBR blend with 2 phr (**a**), 5 phr (**b**) and 10 phr (**c**) nanoclay.

**Figure 8:**
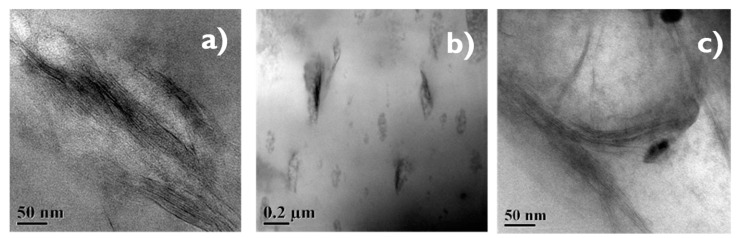
(**a**–**c**) TEM images showing of 30/70 NR/NBR blend with 2, 5 and 10 phr nanoclay.

The effect of incorporating nanoclay into the NR/NBR polymer blend system is shown in Figure 3, Figure 4, Figure 5 and Figure 6, indicating a reduction of the gas permeability of the elastomer films. It is very important to note that the permeability of NBR and NR nanocomposites showed a drastic decrease by the addition a small amount of nanoclay (just 1 wt %), followed by a levelling off. The inorganic platelet morphology of nanoclay makes it impermeable to gases. The large aspect ratio and nanoscale dimensions either in exfoliated stage or intercalated stage present large surface area, even at a low concentration of nanoclay, and thereby reduces the area of cross section available for permeation. The tortuosity also is increased in the blend and hence increases the path length of diffusing molecules. This can be observed from the morphological data from TEM characterization, given in Figure 4 (inset), Figure 7, Figure 8, where the images for different filler loading are provided. The decrease of free volume contribution should be considered too. The decrease in the free volume, due to the densely packed polymer chains [21] as a result of interaction between nanoclay with NR and NBR, can also be the contributing factor in decreasing the permeability. However, it is interesting to note that, for all the NR/NBR blend system with nanoclay, the gas permeability values get an effective decrease only at higher filler loading of 5 phr of clay, while the neat rubbers (NR and NBR) showed a dramatic decrease with just a 1 phr addition of the nanoclay. This can be explained as being based on the availability of nanoclay in creating a barrier effect in blend systems. As evidenced in all the TEM images, in all blend nanocomposites, part of the nanoclay gets localized at the blend interface, which makes decreased availability of nanoclay in the continuous NR and NBR phases to create a strong barrier effect. Therefore, for the development for an effective barrier (network) against gas transport, the system needs more nanoclay—close to 5 phr—to have a significant improvement in barrier effect. As expected, the TEM micrographs show a severe agglomeration of the clay at 10 phr loading (Figure 7c).

### 3.3. Effect of the Gas Nature

**Figure 9:**
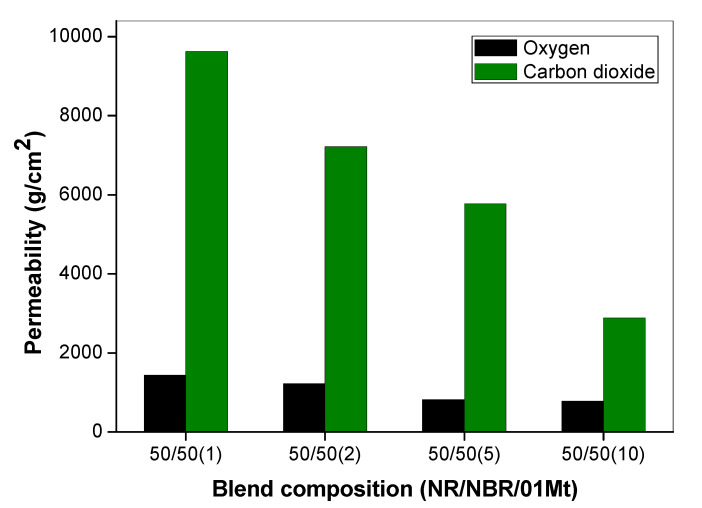
Comparison of oxygen permeability and carbon dioxide permeability of 50/50 NR/NBR blend with varying filler loading.

The effects of the size of gas molecules on the permeability property of 50/50 NR/NBR blend with varying filler loading were also studied and are given in Figure 9. The permeation is basically based on Fick’s Law of diffusion and Henry’s Law of solubilities. The influence of penetrant size clearly contributes to the diffusion of gas molecules. The permeability of the gases showed that the gas transport was not only dependent on the molecular weight, but also on the kinetic diameter of the gases. The molecular mass of O_2_ is 32 and that of CO_2_ is 44 [22]. Though CO_2_ is heavier than O_2_, it is actually smaller in molecular size and can diffuse faster through the polymer. It can be observed that, for all the composition, the permeability of O_2_ is very low compared to CO_2_ (Figure 9). It is interesting to find that the CO_2_ that possess a higher molecular weight is showing higher permeability.

One reason that can be given for this behavior is due to the higher solubility of CO_2_ with rubber. Yet another reason is the size effect. This can be explained using the Stokes-Einstein equation, which explains that the diffusion of gas molecules is inversely related to the friction exerted. The Stokes-Einstein equation is given by:

(2) $$D=K_{B}.T/f$$

where K_B_ is the Boltzmann constant, T is the absolute temperature, and f is the friction factor, which is given by Equation (3).

(3) $$f=6{\pi \mu R}_{O}$$

Kinetic diameters play a major role in gas adsorption and permeation polymeric materials [23]. The kinetic diameter of the two gas molecules also explains this reduction of permeability for O_2_. It is given that for O_2_ the kinetic diameter is 3.4 × 10^−10^ m while for CO_2_ it is 3.3 × 10^−10^ m. The increase in the radius of the gas molecule increases the friction factor by the relation (Equation (3)), and there is a corresponding decrease of permeability. Therefore, the high solubility and low kinetic diameter of CO_2_ explains its faster diffusion than oxygen [24].

### 3.4. Rheological Properties of NR/NBR Blend Nanocomposites

**Figure 10:**
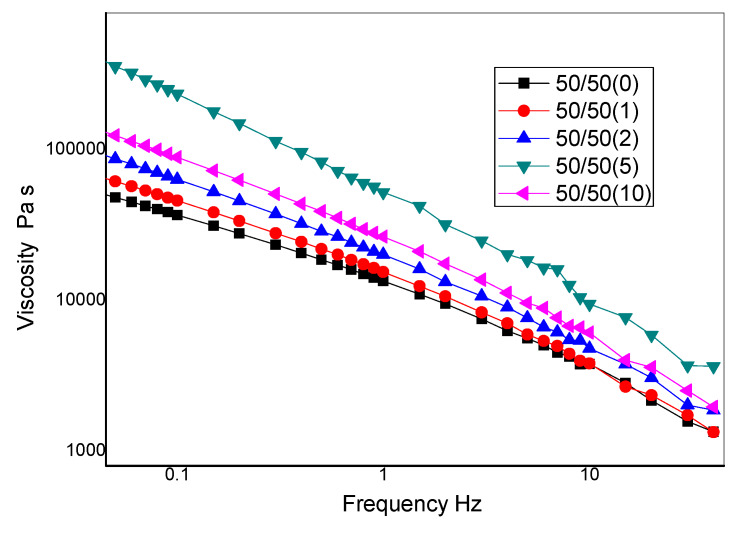
Rheological curves of complex viscosity (η*) vs. frequency for 50/50 NR/NBR blend.

Figure 10 illustrates the effect of nanoclay on the complex viscosity, η*, curves for 50/50 NR/NBR blend. When no shear is applied, the polymer macromolecules show a complex entangled structure which gives rise to the so-called zero shear viscosity. At low shear rates, the complex viscosity of all the filled samples showed a significant increase as compared to that of the neat sample. This is in accordance with earlier reports of theoretical expectations and experimental observations for nanoclay filled elastomer nanocomposites [25,26]. The complex viscosity at the lowest shear rates increases on the increasing of the nanoclay content, indicating the formation of a structural network, but the maximum is observed at a loading of 5 phr. The significant increase in the complex viscosity, can be ascribed to the nanoclay intercalation [27,28] and with the increase of the interfacial surface between the elastomer components and the nanoclay. The decrease of the low shear rate viscosity for the sample with 10 phr of nanoclay may be due to the agglomeration of nanoclay in the blends that gives rise to a strong reduction of the interfacial surface. Figure 10 shows that, at a higher frequency, the complex viscosity strongly decreased, which reveals high shear-thinning behavior of these polymer blends and, in particular, the non-Newtonian behavior, is more pronounced on increasing the content of nanofiller. This behavior can be interpreted in terms of orientation of the nanoplatelets that decreases the resistance to the flow of the solid filler.

### 3.5. Mechanical Properties

**Figure 11:**
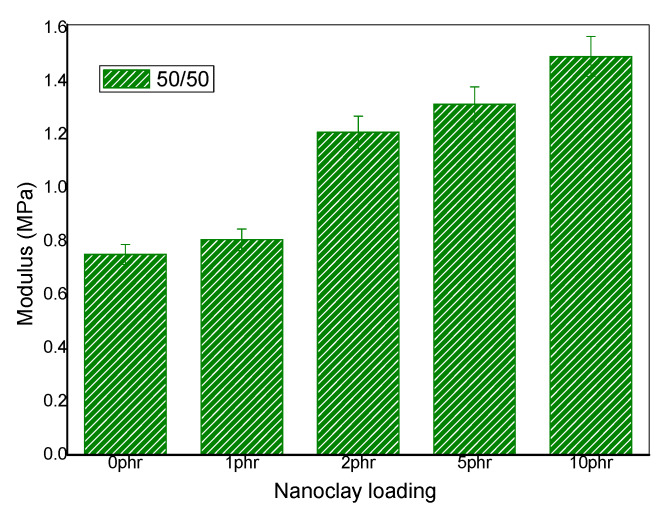
Young modulus (stress at 100%) NR/NBR/nanoclay in 50/50 blend composition.

The effect of nanoclay on the mechanical properties in 50/50 NR/NBR is shown in Figure 11. An improvement in modulus was observed for all the blend nanocomposites in 50/50 NR/NBR blend. This is due to the excellent interaction between rubber chains and the nanoclay. It can be easily observed that the nanoclay gets localized at the interface and at the two phases. Both NR and NBR phases interact with nanoclay, due to surface modification of the clay. The alkyl chains (hydrogenated tallow) on the surface of the clay provides better interaction with the NR phase and the intrinsic polar nature of the clay makes its interaction with NBR phase. Thus, a good polymer-filler interaction contributes toward the improvement in the modulus [29]. However, it is interesting to note that, even though the 10 phr loading shows high degree of agglomeration, as shown by the TEM data, the modulus values were not affected. It is also important to mention that, unlike tensile strength, the modulus is not very much affected by filler/matrix interactions. It is strongly influenced by the filler loading.

## 4. Conclusions

In this work, NR/NBR blends and their blend nanocomposites using Cloisite 10A with 1 phr, 2 phr, 5 phr and 10 phr of nanoclay were prepared. The morphology of the blends and blend nanocomposites were carefully analyzed using TEM and SEM. The addition of NBR to NR dramatically increased the barrier properties, and this could be related to the morphology of the blends. The barrier properties were remarkably improved by the addition of few percent of nanoclay (just 1 phr) into the neat polymers, such as NR and NBR, however, the blends needed a higher amount of clay, due to the interfacial migration of clay. Although the stacks of clay and non-uniform dispersion of clay particle were shown in the TEM micrographs, the tortuosity path for the gas molecules was increased sufficiently to make a significant improvement in gas barrier properties. The diffusion of CO_2_ was much faster than O_2_, on account of the lower kinetic diameter and higher solubility of CO_2_ in the blends. All the blends showed shear thinning behavior, as evidenced by the decrease of viscosity with increase in frequency. The modulus data showed an increase in trend with the addition of fillers.

## Figures and Tables

**Table 1:** The formulation of rubber compounding mixture (curing agents; 100/0,70/30,50/50,30/70 and 0/100 NR/NBR ratios were adopted).

Materials	Curing Agents Parts Per Hundred (phr) of Rubber (in the Order of Mixing)
Zinc oxide	2.5
Stearic acid	1.5
CBS(*N*-cyclohexyl-benzothiazyl sulphonamide)	1.3
TMTD(tetra methyl thiuram disulphide)	0.2
Sulphur	2.25

**Table 2:** Description and identification of the blends and nanocomposites composition.

Blends NR/NBR	Nanocomposites Nanoclay (phr)				
	0	1	2	5	10
100/0	100/0 (0)	100/0 (1)	100/0 (2)	100/0 (5)	100/0 (10)
70/30	70/30 (0)	70/30 (1)	70/30 (2)	70/30 (5)	70/30 (10)
50/50	50/50 (0)	50/50 (1)	50/50 (2)	50/50 (5)	50/50 (10)
30/70	30/70 (0)	30/70 (1)	30/70 (2)	30/70 (5)	30/70 (10)
0/100	0/100 (0)	0/100 (1)	0/100 (2)	0/100 (5)	0/100 (10)

## References

[1] Adrees M., Iqbal S.S., Ahmad A., Jamshaid F., Haider B., Khan M.H., Khan R., Butt M.T.Z., Bahadar A. (2019). Characterization of novel polydimethylsiloxane (PDMS) and copolymer polyvinyl chloride-co-vinyl acetate (PVCA) enhanced polymer blend membranes for CO_2_ separation. Polym. Test..

[2] Matavos-Aramyan S., Jazebizadeh M.H., Babaei S. (2020). Investigating CO_2_, O_2_ and N_2_ permeation properties of two new types of nanocomposite membranes: Polyurethane/silica and polyesterurethane/silica. Nano-Struct. Nano-Objects.

[3] Zembouai I., Kaci M., Bruzaud S., Benhamida A., Corre Y.M., Grohens Y. (2013). A study of morphological, thermal, rheological and barrier properties of Poly (3-hydroxybutyrate-Co-3-Hydroxyvalerate)/polylactide blends prepared by melt mixing. Polym. Test..

[4] Lafitte G., Espuche E., Gérard J.F. (2011). Polyamide 11/poly (hydroxy amino ether) blends: Influence of the blend composition and morphology on the barrier and mechanical properties. Eur. Polym. J..

[5] Subramanian P.M., Mehra V. (1987). Laminar morphology in polymer blends: Structure and properties. Polym. Eng. Sci..

[6] Subramanian P.M., Koros W.J. (1990). Polymer Blends: Morphology and Solvent Barriers, Ch. 13 in Barrier Properties of Polymers. Am. Chem. Soc..

[7] Rooj S., Das A., Thakur V., Mahaling R.N., Bhowmick A.K., Heinrich G. (2010). Preparation and properties of natural nanocomposites based on natural rubber and naturally occurring halloysite nanotubes. Mater. Des..

[8] Boonprasith P., Wootthikanokkhan J., Nimitsiriwat N. (2013). Mechanical, thermal, and barrier properties of nanocomposites based on poly (butylene succinate)/thermoplastic starch blends containing different types of clay. J. Appl. Polym. Sci..

[9] Boudenne A., Mamunya Y., Levchenko V., Garnier B., Lebedev E. (2015). Improvement of thermal and electrical properties of Silicone–Ni composites using magnetic field. Eur. Polym. J..

[10] Kratochvíla J., Boudenne A., Krupa I. (2013). Effect of filler size on thermophysical and electrical behavior of nanocomposites based on expanded graphite nanoparticles filled in low-density polyethylene matrix. Polym. Compos..

[11] Tlili R., Boudenne A., Cecen V., Ibos L., Krupa I., Candau Y. (2010). Thermophysical and electrical properties of nanocomposites based on ethylene–vinylacetate copolymer (EVA) filled with expanded and unexpanded graphite. Int. J. Thermophys..

[12] Frounchi M., Dadbin S., Salehpour Z., Noferesti M. (2006). Gas barrier properties of PP/EPDM blend nanocomposites. J. Membr. Sci..

[13] Yeh J.T., Fan-Chiang C.C., Yang S.S. (1997). Effects of composition of modified polyamide on barrier properties of polyethylene/modified polyamide blends. J. Appl. Polym. Sci..

[14] Yeh J.T., Chang C.J., Tsai F.C., Chen K.N., Huang K.S. (2009). Oxygen barrier and blending properties of blown films of blends of modified polyamide and polyamide-6 clay mineral nanocomposites. Appl. Clay Sci..

[15] Ghanbari A., Heuzey M.C., Carreau P.J., Ton-That M.T. (2013). Morphological and rheological properties of PET/clay nanocomposites. Rheol. Acta.

[16] Bitinis N., Verdejo R., Maya E.M., Espuche E., Cassagnau P., Lopez-Manchado M.A. (2012). Physicochemical properties of organoclay filled polylactic acid/natural rubber blend bionanocomposites. Compos. Sci. Technol..

[17] Kuriakose B., De S.K. (1985). Studies on the melt flow behavior of thermoplastic elastomers from polypropylene—natural rubber blends. Polym. Eng. Sci..

[18] Meares P. (1954). The diffusion of gases through polyvinyl acetate1. J. Am. Chem. Soc..

[19] Alentiev A.Y., Yampolskii Y.P. (2002). Meares equation and the role of cohesion energy density in diffusion in polymers. J. Membr. Sci..

[20] Kubica P., Wolinska-Grabczyk A. (2015). Correlation between cohesive energy density, fractional free volume, and gas transport properties of poly (ethylene-co-vinyl acetate) materials. Int. J. Polym. Sci..

[21] Yang Y.H., Haile M., Park Y.T., Malek F.A., Grunlan J.C. (2011). Super gas barrier of all-polymer multilayer thin films. Macromolecules.

[22] Studymate. https://www.studymateonline.com/media/filer_public/65/6e/656ed9e6-5f92-45ff-9382-3ce08b07ada6/ch_1_11th_some_basic_concept.pdf.

[23] Mehio N., Dai S., Jiang D.E. (2014). Quantum mechanical basis for kinetic diameters of small gaseous molecules. J. Phys. Chem. A.

[24] Chaix E., Guillaume C., Guillard V. (2014). Oxygen and carbon dioxide solubility and diffusivity in solid food matrices: A review of past and current knowledge. Compr. Rev. Food Sci. Food Saf..

[25] Cassagnau P. (2008). Melt rheology of organoclay and fumed silica nanocomposites. Polymer.

[26] Meera A.P. (2010). Effect of Spherical and Layered Type Fillers on the Morphology and Physico Mechanical Properties of Natural Rubber Nanocomposites. Ph.D. Thesis.

[27] Maria H.J., Lyczko N., Nzihou A., Joseph K., Mathew C., Thomas S. (2014). Stress relaxation behavior of organically modified montmorillonite filled natural rubber/nitrile rubber nanocomposites. Appl. Clay Sci..

[28] La Mantia F.P., Mistretta M.C., Morreale M. (2014). Recycling and thermomechanical degradation of LDPE/modified clay nanocomposites. Macromol. Mater. Eng..

[29] Srivastava S.K., Mishra Y.K. (2018). Nanocarbon reinforced rubber nanocomposites: Detailed insights about mechanical, dynamical mechanical properties, Payne, and Mullin effects. Nanomaterials.

